# Ethnic minority inequalities in access to treatments for schizophrenia and schizoaffective disorders: findings from a nationally representative cross-sectional study

**DOI:** 10.1186/s12916-018-1035-5

**Published:** 2018-04-18

**Authors:** Jayati Das-Munshi, Dinesh Bhugra, Mike J. Crawford

**Affiliations:** 1 Institute of Psychiatry, Psychology and Neuroscience, King’s College London, London, UK; 2 Centre for Psychiatry, Imperial College London, London, UK; 3 South London & Maudsley NHS Foundation Trust, London, UK

**Keywords:** Inequalities, Race, Ethnic minority, Schizophrenia, Schizoaffective disorders, Treatments, Prescribing, CBT, Family therapy, Care plans

## Abstract

**Background:**

Ethnic minority service users with schizophrenia and schizoaffective disorders may experience inequalities in care. There have been no recent studies assessing access to evidence-based treatments for psychosis amongst the main ethnic minority groups in the UK.

**Methods:**

Data from nationally representative surveys from England and Wales, for 10,512 people with a clinical diagnosis of schizophrenia or schizoaffective disorders, were used for analyses. Multi-level multivariable logistic regression analyses were used to assess ethnic minority inequalities in access to pharmacological treatments, psychological interventions, shared decision making and care planning, taking into account a range of potential confounders.

**Results:**

Compared with white service users, black service users were more likely prescribed depot/injectable antipsychotics (odds ratio 1.56 (95% confidence interval 1.33–1.84)). Black service users with treatment resistance were less likely to be prescribed clozapine (odds ratio 0.56 (95% confidence interval 0.39–0.79)). All ethnic minority service users, except those of mixed ethnicity, were less likely to be offered cognitive behavioural therapy, compared to white service users. Black service users were less likely to have been offered family therapy, and Asian service users were less likely to have received copies of care plans (odds ratio 0.50 (95% confidence interval 0.33–0.76)), compared to white service users. There were no clinician-reported differences in shared decision making across each of the ethnic minority groups.

**Conclusion:**

Relative to white service users, ethnic minority service users with psychosis were generally less likely to be offered a range of evidence-based treatments for psychosis, which included pharmacological and psychological interventions as well as involvement in care planning.

## Background

There have been longstanding concerns around the care which ethnic minority groups living with severe mental illnesses receive [1–3]. In particular, it is well documented that ethnic minority groups, and in particular black people with psychosis, are more likely to experience complex [1] and coercive pathways into care [1, 4]. Concerns around coercive practices have led some to consider the role of prescribing inequalities by ethnicity, as prescribing practices may reflect discriminatory practices. A recent systematic review of studies, mostly from the USA, suggested that ethnic minority groups were more likely to be prescribed typical antipsychotics over atypical antipsychotics [5]. Evidence from the UK has not indicated differences in prescribing quality by ethnicity; however, these studies have been based on in-patient populations, with relatively small numbers and have only assessed differences between black and white patients [6, 7]. It is possible that inequalities in treatments may be a concern for other ethnic minority groups as well.

There is less evidence on access to psychological treatments for schizophrenia in black and minority ethnic groups. People with schizophrenia should be offered cognitive behavioural therapy (CBT) or family therapy [8], and from recent data, how far this quality standard is met within ethnically diverse populations remains unclear. Previous research, from almost a decade ago, suggested that black Caribbean people with psychosis were less likely to receive psychotherapy [9], and Asian and black service users under the care of community mental health teams were also less likely to be referred for psychological treatments [3]. It is possible that there have been changes since the introduction of the recent guidelines for schizophrenia [8], but no up-to-date studies have assessed this.

Alongside the receipt of treatments for schizophrenia, service users with schizophrenia should be at the heart of decision making. To this end, the choice of antipsychotic medication should be determined through discussion between the clinician and the service user [8, 10], information relating to potential side effects alongside benefits should be clear [8, 10] and written information or an equivalent should be given to service users with schizophrenia and schizoaffective disorders [8, 10]. There should be a care plan jointly agreed on by the clinician and service user and, if appropriate, with the involvement of carers [8, 10]. It is not yet clear how far standards relating to these aspects of shared decision making fall short for ethnic minority groups with schizophrenia.

We conducted a secondary analysis of nationally representative data of people living with schizophrenia and schizoaffective disorders, to determine whether there were differences in treatments (antipsychotic prescribing, psychological therapies, shared decision making and receipt of care plans) across ethnic minority groups.

## Methods

### Setting and participants

The National Audit of Schizophrenia (NAS) was a cross-sectional survey of all mental healthcare trusts and health boards within England and Wales that aimed to collect information on randomly selected patients from healthcare providers on the quality of care provided. In the UK, mental health trusts provide mental healthcare to geographically distinct catchment areas and provide both in-patient and out-patient services; they may also occasionally provide tertiary-level services at the national level. Despite being separate mental health providers, trusts are still expected to provide equivalent standards of care nationally. There have been two waves of data collection for the surveys, first in 2011 [11] and then in 2013 [12]. On each occasion, trusts were asked to provide data on a random sample of 100 service users over the age of 18 with a diagnosis of ICD-10 F20.0-F20.9 (schizophrenia) or F25.0-25.9 (schizoaffective disorder) under the care of community-based services (community mental health teams and specialist teams) within the previous 12 months, with the diagnosis made before the age of 60 years. Patients on in-patient caseloads were excluded. Selection of patients for both surveys was based on established random selection techniques, which included an online randomisation tool or randomly generated numbers by the study team, with mental health trusts generating a sampling frame which comprised case lists from teams or selecting cases centrally using online random selection techniques. Once service users were identified using these methods, local staff were contacted and asked to fill out the data collection questionnaire. They were also able to refer to clinical case notes as well as consult with the service users’ general practitioners, if required. Service providers submitted the completed questionnaires via a secure online system. All trusts were asked to verify if data were accurate prior to analysis. A priori sample size calculations determined the number of returns needed in order to have adequate power to provide sufficient precision of proportions to make meaningful comparisons between services. All responses were anonymised and kept confidential. Full details of the methods for both surveys, as well as the extensive piloting phase, have been reported elsewhere [11, 12].

### Data collection procedures

Prior to conducting the first survey, focus groups were conducted, alongside a development phase for outcome indicators. A pilot study comprising six trusts was conducted and further helped to inform methods of data collection. The resultant questionnaire took less than 20 min to complete. The final tool included specific measures designed to assess quality of care (prescribing, access to psychological treatments and receipt of physical healthcare monitoring and interventions) according to standards set through the National Institute for Health and Care Excellence (NICE) for the management of people with schizophrenia [8]. The findings related to physical health have been published elsewhere [13].

### Measures

The surveys were designed to assess if standards were being met in the quality of care received by people with schizophrenia and schizoaffective disorders, according to national quality standards [11, 12]. Service users and providers of mental health services formed an expert reference group and were consulted in the development of the questionnaires. Across the two waves of data collection, questionnaires and instruments which had been successfully piloted and used in the first wave were kept similar to those employed in wave two. Except for a question on whether clinicians had provided service users with a care plan (asked only in the second wave of the survey, as this was not a national standard when the first survey was conducted), virtually identical information on all other indicators was collected across the two waves.

Trusts were asked to provide information on currently prescribed antipsychotic medication and to list all currently prescribed antipsychotic medications with dose. Where service users were not currently prescribed antipsychotic medication, trusts were asked to report details relating to most recently prescribed medications. Information relating to current/recent antipsychotic prescribing (excluding *pro re nata* (PRN) medications), in particular relating to type, dose and method of administration, were collected. Measures for maximum doses reached (as percentage of recommended maximum doses according to British National Formulary guidelines [14]) and evidence of concurrent prescribing of more than one antipsychotic medication at the same time were also noted from the responses given to the question asking about currently prescribed antipsychotic medications. Evidence of concurrent prescribing of more than one antipsychotic medication at the same time was also noted from the responses given to the question asking about currently prescribed antipsychotic medications. Using this information, a variable was created to identify individuals prescribed more than one antipsychotic medication at the same time, excluding clozapine. The name provided for each of the antipsychotic medications was used to determine the type of antipsychotics (first generation, second generation, clozapine). The method of administration (oral, depot) was also noted. Clinicians were asked whether they thought the patient was in remission and, if not, the level of disability evident. Where clinicians reported that a patient was not in remission or had only partial remission with significant symptoms and disability, they were asked if clozapine had been offered. Evidence of prior or current psychological intervention ever being offered was noted (family therapy and CBT). Service providers were also asked to report whether there was documented evidence that service users had been involved in deciding which antipsychotic medication should be prescribed, whether there was documented evidence in the notes that the service user had been provided with written information on medication or an alternative, and whether the benefits and the side effects of the medication had been explained.

Trusts were asked to extract data on ethnicity from clinical records and indicate whether the service user was ‘White’, ‘Asian or Asian British’, ‘Black or Black British’, ‘Chinese’ or ‘Other’ ethnic group, ‘Mixed’ or ‘Not stated’. These categories were retained for the analysis.

Information on other socio-demographic indicators, including gender, age and team responsible for care (assertive outreach team, community mental health team, crisis resolution team, early intervention and ‘other’ teams) and information on diagnoses according to the International Classification of Mental Disorders-10 (ICD-10) [15] was collected, as well as duration of illness.

### Statistical analysis

Simple descriptive analyses were initially conducted on each of the two datasets using one-way analysis of variance (ANOVA) and χ^2^ tests as appropriate. Multi-level random effects logistic regression models were then used to assess the association of individual-level covariates, nested within mental health trusts, with each of the treatment outcomes. These models specifically modelled the variance between ‘clusters’ (in this case trusts) as well as within clusters. Each of the individual-level variables (age, sex, ethnicity, ICD-10 diagnosis, duration of illness, team providing care, remission status) were modelled as fixed effects, with ethnicity treated as the main exposure and all other variables as a priori confounders. For each model the likelihood ratio test (LRT) for ‘rho’ or the intra-cluster correlation coefficient (ICC) assessed clustering within the dataset against a null hypothesis of no clustering. A statistically significant *p* value from the LRT indicates evidence of within-trust clustering. Using this approach, the crude association of ethnicity with each of the treatment outcomes was derived, followed by the association of ethnicity with each of the treatment outcomes adjusted for a priori confounders. For analyses relating to clozapine prescriptions, models were re-run with the sample restricted to individuals who were not in remission or had only partial remission with significant disability. Prior to combining waves of the NAS, an interaction term survey year*ethnicity was fitted in models for all outcomes. As there was no evidence in support of a statistical interaction, analyses were combined across the 2 years. All analyses were conducted in STATA 13 [16].

## Results

At trust level, for the first wave, 60/64 (94%) of mental health trusts provided data, and for the second wave, all 64/64 mental health trusts in England and Wales submitted data. At an individual level, response rates were 85% in the first wave and 88% in the second wave.

Table 1 highlights demographic features of the sample. In general, ethnic minority service users within the sample were younger than white service users, with a similar sex profile. Service users who were in the Chinese, ‘Other’ or ‘Mixed’ ethnicity groups were more likely to be diagnosed as having a schizoaffective disorder, whilst duration of illness was longer in the white group. A higher proportion of black/black British service users were under the care of an assertive outreach team.

**Table 1:** Demographic features by ethnicity

	Full sample		White		Asian/Asian British		Black/Black British		Chinese/ Other		Mixed		
	*N*	(%)	*N*	(%)	*N*	(%)	*N*	(%)	*N*	(%)	*N*	(%)	ANOVA/ χ^2^
Total	10,504		8368		861		883		183		209		
Mean age in years (SD)			46.7	(13.4)	42.0	(12.2)	43.0	(11.9)	45.4	(13.7)	38.7	(12.0)	*F* = 52.6; *p* < 0.001
Sex													
Male	6834	65%	5463	65%	538	62%	570	65%	117	64%	146	70%	χ^2^ = 5.01; *p* = 0.29
Female	3670	35%	2905	35%	323	38%	313	35%	66	36%	63	30%	
Diagnosis													
F20 (schizophrenia)	8854	84%	7010	84%	743	86%	778	88%	147	80%	176	84%	χ^2^ = 16.21; *p* = 0.003
F25 (schizoaffective disorder)	1650	16%	1358	16%	118	14%	105	12%	36	20%	33	16%	
Duration of illness													
1–2 years	466	4%	330	4%	51	6%	55	6%	14	8%	16	8%	χ^2^ = 123.69; *p* < 0.001
2–4 years	961	9%	695	8%	106	12%	107	12%	22	12%	31	15%	
4–10 years	2607	25%	1982	24%	270	31%	252	29%	49	27%	54	26%	
10+ years	6470	62%	5361	64%	434	50%	469	53%	98	54%	108	52%	
Level of remission													
Full/partial	7711	73%	6168	74%	617	72%	633	72%	137	75%	156	75%	χ^2^ = 3.44; *p* = 0.49
Significant disability or no remission	2793	27%	2200	26%	244	28%	250	28%	46	25%	53	25%	
Clinical team responsible for care													
Community mental health team	7435	71%	6062	72%	597	69%	532	60%	131	72%	113	54%	χ^2^ = 137.93; *p* < 0.001
Assertive outreach team	1294	12%	1004	12%	93	11%	143	16%	14	8%	40	19%	
Early intervention	520	5%	338	4%	66	8%	75	8%	14	8%	27	13%	
Other incl. crisis resolution team	1255	12%	964	12%	105	12%	133	15%	24	13%	29	14%	

Table 2 displays proportions accessing treatments for psychosis by ethnicity. The majority of service users (96% in full sample) were prescribed an antipsychotic medication. White service users and people of mixed ethnicity were more likely to exceed recommended BNF dose limits, and a larger proportion of black/black British service users were prescribed a depot. For psychological treatments, Asian/Asian British service users were more likely to be referred to family therapies, while service users of Chinese/other ethnicity were less likely to have documented evidence of their being given a copy of their care plan.

**Table 2:** Receipt of treatments by ethnicity

	Full sample		White		Asian/ Asian British		Black / Black British		Chinese/ Other		Mixed		
	*N*	(%)	*N*	(%)	*N*	(%)	*N*	(%)	*N*	(%)	*N*	(%)	*χ*^*2*^*; p* value
Totals	10,504		8368		861		883		183		209		
Antipsychotic prescribing													
In receipt of an antipsychotic	10,111	96%	8068	96%	828	96%	839	95%	174	95%	202	97%	*χ*^2^ = 5.16; *p* = 0.27
Prescribed SGA vs FGA^a^	4396	65%	3362	64%	428	70%	418	63%	97	73%	91	68%	*χ*^2^ = 13.41; *p* = 0.009
Prescribed depot	3336	32%	2640	32%	257	30%	335	38%	46	25%	58	28%	*χ*^2^ = 22.4; *p* < 0.001
Prescribed clozapine^b^	719	26%	589	27%	57	23%	46	18%	12	26%	15	28%	*χ*^2^ = 9.18; *p* = 0.06
Prescribed > 100% BNF recommended dose	1048	10%	869	10%	69	8%	65	7%	16	9%	29	14%	*χ*^2^ = 15.82; *p* = 0.003
Prescribed 2+ antipsychotics, exc. clozapine	1229	12%	1019	12%	91	11%	78	9%	16	9%	25	12%	*χ*^2^ = 11.50; *p* = 0.02
Shared decision making													
Service user involved in deciding which antipsychotic prescribed	6070	59%	4861	59%	470	55%	514	59%	99	55%	126	62%	*χ*^2^ = 6.17; *p* = 0.19
Provided with written info on medication or alternative	4244	41%	3373	40%	349	41%	367	42%	71	39%	84	41%	*χ*^2^ = 0.69; *p* = 0.95
Benefits/side effects of the antipsychotic explained	7421	71%	5885	71%	604	70%	650	74%	125	69%	157	76%	*χ*^2^ = 6.94; *p* = 0.14
Psychological treatments													
CBT ever offered	3578	34%	2826	34%	276	32%	325	37%	57	31%	94	45%	*χ*^2^ = 16.60; *p* = 0.002
Family therapy ever offered	1554	15%	1176	14%	187	22%	114	13%	36	20%	41	20%	*χ*^2^=46.35; *p* < 0.001
Care plan^c^													
Service user has a current care plan	5263	95%	4208	96%	408	91%	435	96%	98	91%	114	98%	*χ*^2^ = 24.34; *p* < 0.001

^a^Second generation antipsychotic (SGA) vs first generation antipsychotic (FGA), excluding people prescribed either clozapine, both FGA and SGA or no antipsychotics; totals: full sample *n* = 6775, White *n* = 5237, Asian/Asian British *n* = 612, Black/Black British *n* = 661, Chinese/Other *n* = 132 Mixed *n* = 1331

^b^Clozapine prescribing restricted to *n* = 2793 people not in remission (partial remission with substantial disability or not in remission at all)

^c^Asked in second wave only

Table 3 displays adjusted odds ratios for the association of ethnicity with each of the treatment indicators from multi-level multivariable logistic regression models. Of note, LRTs which assessed the null hypothesis of no within-trust clustering vs the alternative hypothesis of some within-trust clustering (i.e. LRT for rho = 0, also known as the ICC) were *p* < 0.001 for each of the treatment indicators (antipsychotic prescribing, referrals for psychological therapies, shared decision making and whether service users had a care plan) displayed in Table 3. This suggests that high levels of variance remained between National Health Service (NHS) mental health trusts after adjusting for all individual-level covariates, which may indicate that certain NHS mental health trust-level characteristics might account for variability in treatment provision, despite adjustment for each of the individual-level covariates in the models.

**Table 3:** Receipt of treatments by ethnicity

		Asian/Asian British		Black/Black British		Chinese/ Other		Mixed		LRT
	Number, *N*/ total	OR	95% CI	OR	95% CI	OR	95% CI	OR	95% CI	*p* value
Antipsychotic prescribing										
Not in receipt of an antipsychotic	393/10,504	1.01	0.69, 1.47	1.26	0.89, 1.77	1.24	0.62, 2.49	0.78	0.36, 1.70	0.66
Prescribed SGA vs FGA^a^	4396/6769	0.91	0.75, 1.10	1.17	0.97, 1.41	0.72	0.48, 1.07	1.06	0.72, 1.56	0.13
Prescribed depot	3336/10,504	1.07	0.90, 1.26	1.56	1.33, 1.84	0.83	0.59, 1.17	1.01	0.74, 1.40	< 0.001
Prescribed clozapine^b`^	719/2793	0.76	0.55, 1.06	0.56	0.39, 0.79	0.96	0.48, 1.92	1.10	0.58, 2.08	0.01
Prescribed > 100% BNF recommended dose	1048/10,504	0.82	0.63, 1.08	0.77	0.58, 1.02	0.88	0.52, 1.50	1.58	1.05, 2.38	0.04
Prescribed 2+ antipsychotics, exc. clozapine	1229/10,504	1.00	0.79, 1.28	0.86	0.66, 1.11	0.77	0.46, 1.31	1.20	0.79, 1.86	0.54
Shared decision making										
Service user involved in deciding which antipsychotic prescribed	6070/10,334	0.85	0.73, 0.99	1.07	0.92, 1.26	0.93	0.68, 1.27	1.08	0.80, 1.45	0.17
Provided with written info on medication or alternative	4244/10,455	0.97	0.83, 1.14	1.04	0.88, 1.22	0.96	0.70, 1.32	0.94	0.70, 1.27	0.96
Benefits/side effects of the antipsychotic explained	7421/10,461	0.93	0.79, 1.10	1.15	0.96, 1.37	0.97	0.70, 1.35	1.20	0.86, 1.68	0.31
Psychological treatments										
CBT ever offered	3578/10,495	0.73	0.61, 0.86	0.74	0.63, 0.88	0.69	0.49, 0.97	1.10	0.82, 1.48	< 0.001
Family therapy ever offered	1554/10,493	1.53	1.26, 1.86	0.76	0.60, 0.95	1.39	0.93, 2.06	1.01	0.70, 1.47	< 0.001
Care plan^c^										
Service user has a current care plan	5263/5520	0.50	0.33, 0.76	1.29	0.74, 2.26	0.53	0.26, 1.10	2.38	0.56, 10.12	0.002

Reference group for all analyses is the ‘White’ ethnicity group

All models adjust for age, sex, managing team, duration of illness, diagnosis, remission and NAS survey year

^a^Excludes people prescribed combination of drugs, no drugs or clozapine

^b^Clozapine restricted to people not in remission (partial remission with substantial disability or not in remission at all)

^c^Second wave only

*LRT* likelihood ratio test, *OR* odds ratio, *CI* confidence interval, *SGA* second generation antipsychotic, *FGA* first generation antipsychotic, *BNF* British National Formulary, *NAS* National Audit of Schizophrenia

In adjusted models, relative to white service users with schizophrenia and schizoaffective disorders, each of the ethnic minority groups, except people who were of mixed ethnicity, were less likely to exceed BNF maximum doses for antipsychotic prescribing, although service users of mixed ethnicity were more likely to experience high-dose prescribing relative to white service users (Table 3). Black/black British service users were more likely to be prescribed depot antipsychotics compared to white service users. When analysis of clozapine was restricted to the sample of people with treatment-resistant schizophrenia, black/black British service users were less likely to be prescribed clozapine relative to white service users, with a trend towards a similar direction in the Asian/Asian British group. In adjusted regression models, there was no evidence of an association between ethnicity and antipsychotic polypharmacy. There were no reported differences by ethnicity with respect to clinicians reporting that they had provided service users with written information on medications, had explained the benefits or side effects of the most recent antipsychotic medication, or had involved patients in decisions around the choice of antipsychotic (Table 3).

With respect to psychological therapies, in adjusted models, each of the ethnic minority groups except for the mixed ethnicity group were less likely to have been offered CBT relative to the white group. The Asian/Asian British users were more likely to have been offered family therapy, whereas black/black British service users were less likely to have been offered family therapy, compared to white service users. Asian/Asian British service users were less likely to have been given a copy of their care plans compared to white service users.

## Discussion

The findings from this nationally representative survey of schizophrenia and schizoaffective disorders suggest large variations in the provision and offer of high quality treatments to ethnic minority service users compared to white service users. Differences were observed across the provision of antipsychotic medications, psychological therapies and care plans. Our findings also indicated that variability in estimates remained at trust level even after adjusting for individual-level attributes. This may indicate a need to consider systemic/institutional factors, and could also include an assessment of area-level deprivation or own ethnic density, alongside individual-level factors in accounting for observed ethnic differences, in future work. We did not have trust-level information to inform our analyses, and as such, residual confounding by trust-level factors may be a concern. Future waves of the surveys and subsequent analyses could include trust-level information (such as ethnic diversity, trust size, trust-level policies focusing on ethnic minority disparities) which may inform future work in this area.

The data for this study were collected prior to the introduction of the updated (2014) UK NICE guidelines for the management of schizophrenia [8] and were based on the standards set out in the earlier guidelines (2009). It is possible that since the introduction of the updated guidance, disparities may have improved; future planned sweeps of the NAS will be well placed to monitor this.

### Strengths and limitations

This analysis derives from randomly selected records of service users with schizophrenia and schizoaffective disorders from all mental health trusts in England and Wales. Across mental health trusts, response rates were good. Therefore, the findings are highly generalisable to individuals with these diagnoses, within these countries. Combining two waves of survey meant that more than 10,000 service users provided data for the analysis. This would have improved the statistical power to detect differences. It was possible to survey a variety of treatments for schizophrenia and schizoaffective disorders, and the analyses were not just restricted to prescribing. The findings provide some indication of quality of care received by ethnic minorities with schizophrenia and schizoaffective disorders under the care of community outpatient teams.

One of the limitations of the study is that data were extracted from clinical records. It is possible that in some instances these records did not provide a complete picture of the care that people received. For instance, some patients may have been offered a psychological therapy or a copy of a care plan without this being documented in their notes.

Race/ethnicity is a complex multi-faceted construct, with the two terms frequently used interchangeably across the international literature [17, 18]. Typically ‘race’ has been taken to refer to differences based on biological attributes, and ‘ethnicity’ reflects the way in which individuals place themselves with respect to shared identity, cultural/religious affiliations and positions of marginality [17]. As such, ethnicity may be assessed through self-report, but even here may be subject to change over time, and is not a static construct. Arguably both race and ethnicity are social constructs, subject to change, and potentially capturing systems of oppression [18]. In this study we have preferred to use the terminology ‘ethnicity’ to reflect the complexities of categorisation [19]. A limitation of this study was that we do not know how each of the trusts collected this information on service users. It is possible that for at least some of the service users within the survey, ethnicity was not self-ascribed and was instead recorded by administrators or clinicians using visual attributes. In addition we only had relatively crude indicators for ethnicity, and it is likely that differences between groups were masked. The ‘White’ group would have potentially included individuals of Irish ethnicity as well as other white ethnic groups. It is a limitation that we were unable to explore this further, given concerns around known mental health inequalities affecting these groups [20, 21], as well as a well-established association between migration in general and schizophrenia [22]. In addition, the other ethnic groups (‘Black’, ‘Asian’, ‘Chinese’, ‘Other’, ‘Mixed’) would have contained individuals with differing countries of birth, language, migration and settlement histories, and we were unable to assess these potential indicators of acculturation and migration further.

Although we adjusted for a number of important variables in models, we did not have information on socio-economic position. Information on other clinical factors was also unavailable, including the presence of other co-morbid conditions. It is possible that some of the estimates were confounded by these factors. We were able to assess the role of clustering at the trust level in accounting for variance, but we did not have any trust-level attributes which could be added to the analyses. The role which systemic factors may play in accounting for the differences (such as trust-level commitment to delivering race equality, the financial status of mental health trusts and associated availability of treatments and interpreters) may play a role in patterning differences in outcomes and could be investigated in future work [23].

### Implications

#### Prescribing differences

Previous research has indicated that black people may be more likely to be subjected to community treatment orders (CTOs) (supervised treatment orders with stipulated conditions), for whom depot/injectable prescriptions are also more likely [24]. In addition, in another study, participants prescribed depot formulations experienced and reported the depot to be more coercive and less empowering than people prescribed oral medications [25]. These findings do raise a potential concern around differing experiences of coercion across ethnic minority groups, but should also be considered against the potential harm/benefits of depot. For example, in a recent systematic review and meta-analysis, the authors highlighted that depot preparations were more likely to reduce the risk of relapse compared to oral preparations [26]. It is also possible that preferences for injectable medications follow differing cultural expectations for allopathic treatments [27]. It is possible that the frequency of depot prescriptions by ethnicity observed in the present study reflected these and other factors, although we were unable to assess this further. Future research, potentially utilising qualitative methods, could explore this topic.

Observed differences in antipsychotic dosing may have reflected differing sensitivities (e.g. to extrapyramidal side effects) to antipsychotic medications as well as differences in response to relatively lower doses of antipsychotics by ethnicity, within the sample [28, 29], alongside possible prescriber concerns regarding pharmacokinetic interactions. Differences in dose of antipsychotics may have been mediated through a number of factors including but not limited to tobacco use, dietary factors, illness behaviours, patient expectation/adherence, age, gender and weight [28–30], alongside enzyme-mediated differences in metabolism [29]. We were only able to adjust for some of these factors in our models. Research within this area is still relatively scant [28], specifically with respect to the role of cultural factors in shaping expectation and response to treatments [27, 28]. More could be done to understand this better.

Clozapine is recommended for treatment-resistant schizophrenia, where two or more previous antipsychotic medications have failed to control symptoms [8]. Clozapine has also been associated with a reduction in mortality from natural causes [31] and is associated with a lower all-cause and suicide mortality risk, relative to other antipsychotic medications [32]. The benefits of clozapine on mortality risk are not just due to frequent contacts with health services [31]. A previous study from the UK did not find an association between ethnicity and theoretical delay in initiating clozapine [33], although studies from the USA have indicated that black and Hispanic Americans with treatment-resistant psychosis were less likely to be commenced on clozapine than white Americans [34]. The findings from our analyses suggest that, despite similar proportions of individuals by ethnicity experiencing significant disability or only partial remission from symptoms, the black/black British group and to an extent the Asian/Asian British group were less likely to be prescribed clozapine. The larger sample size and national representativeness of our sample may account for the differences between this and previous UK studies [33, 35]. Ethnic minority disparities in clozapine use may be due to clinician concerns around potential side effects, including agranulocytosis, weight gain and type 2 diabetes mellitus [34]. The reasons for differences in clozapine prescriptions by ethnicity for treatment-resistant psychosis will require further exploration in future work.

#### Psychological treatments

Healthcare providers fell short of recommended guidelines for the management of schizophrenia [8] in ethnic minority groups. Although there are concerns that the wholesale imposition/application of psychological therapies insensitive to cultural context may be Eurocentric, culturally adapted therapies have a role to play in the treatment of psychosis across cultures. Psychological therapies may help to improve therapeutic alliance, adherence to medication and insight, and may help to provide knowledge about a condition and therefore potentially address stigma [36].

Although previous work has indicated increased dropout rates from clinical trials of insight-focused CBT in psychosis in African Caribbean and black African service users [37], the question within the present study assessed whether CBT or family therapies had *ever been offered,* potentially indicating a belief by service providers that some of the ethnic groups, except for the mixed group, were less suitable for CBT than white service users with schizophrenia. CBT when culturally adapted is beneficial for the treatment of psychosis across cultures [37]. Of those studies which have included ethnic minorities in trials of CBT for psychosis, no differences in response have been found for treatment-resistant schizophrenia by ethnicity [38] or in satisfaction with therapy [39]. In a recent clinical trial assessing culturally adapted CBT for psychosis in black British, African Caribbean/black African and South Asian Muslim individuals, the investigators noted that CBT was beneficial for the reduction of positive symptoms and depressed mood [37].

Whereas Asian/Asian British people (and to an extent Chinese/‘Other’ groups) were more likely than white service users to have been offered family therapy, this was less the case for black/black British individuals within the survey. Family therapy may be more acceptable to people from collectivistic cultures [40] and easier to deliver if individuals are in close contact with their families. A randomised controlled trial for family therapy in black, Hispanic and white Americans indicated that culturally adapted family interventions were beneficial for psychotic symptoms compared to a psychoeducational control [41]. It was not possible to assess reasons for the variability in referral practices to family therapy by ethnicity within the study, although the lower rates of referral for the black/black British group will require further exploration.

#### Care plans

Care plans form the core of high quality care and should be developed jointly between the service user and clinician, with a copy of the care plan given to the service user [42]. The care plan should include a psychiatric and psychological formulation and also detail aspects of physical health [42]. An important aspect of the care plan is to help promote socially inclusive opportunities (such as employment). Although, in general, high proportions across the sample (> 91%) had a copy of their care plan relative to white service users, Asian/Asian British service users were less likely to have received a copy of their care plan. This finding is consistent with a previous national patient satisfaction survey conducted more than a decade ago in the UK which also indicated low levels of satisfaction amongst Asian service users, for example with respect to being well informed/having information on mental health services [3]. The findings in the current study highlight a need to ensure that basic standards of care are accessible in an equitable manner across all ethnic minority groups.

#### Trust-level variation

Finally, it is possible that trust-level factors play an important role in determining variations in the treatment of psychosis by ethnicity [23]. Although our analyses hinted at this, we were unable to assess this further. Future research could explore potential systemic/institutional factors alongside individual factors which may impact on access to high quality treatments for psychosis in ethnic minority groups.

## Conclusion

In conclusion, the findings of this analysis of data from a nationally representative survey of service users with schizophrenia and schizoaffective disorders indicate ethnic minority disparities in psychotropic medication use and access to psychological treatments and care plans, in England and Wales. Greater efforts need to be made to ensure that people with psychosis receive interventions and treatments in an equitable manner. Further rounds of the audit planned for the coming years will provide evidence of progress being made, to ensure this at both local and national levels.

## Abbreviations

BNF: British National Formulary
CBT: Cognitive behavioural therapy
CTO: Community treatment order
ICC: Intra-cluster correlation coefficient
ICD-10: International Classification of Diseases 10th edition
LRT: Likelihood ratio test
NHS: National Health Service
PRN: Pro re nata
UK: United Kingdom
